# Political polarization may affect attitudes towards vaccination. An analysis based on the European Social Survey data from 23 countries

**DOI:** 10.1093/eurpub/ckae002

**Published:** 2024-01-26

**Authors:** Michał Wróblewski, Andrzej Meler

**Affiliations:** Institute of Sociology, Nicolaus Copernicus University in Toruń, Toruń, Poland; Institute of Sociology, Nicolaus Copernicus University in Toruń, Toruń, Poland

## Abstract

**Background:**

Political polarization may result in increased confirmation bias, strengthening of disinformation mechanisms or policy distortion. This raises the question whether it may influence the vaccination programmes. This study investigates the relationship between the level of political polarization and coronavirus disease 2019 vaccine behaviour and vaccine acceptance.

**Methods:**

In our study, we use the Partisan Polarization Index (PPI). The PPI aims to measure the gap between satisfaction with the government expressed by supporters of the ruling parties and that expressed by supporters of the opposition parties. We use the data from the latest round of the European Social Service from 23 countries. The effect of the PPI on the variability of vaccination declarations and intent across countries was verified based on a linear regression model.

**Results:**

The linear regression score for 23 countries revealed a statistically significant relationship between the vaccination variable and the level of political polarization. The model explains the variability in the vaccination levels based on the PPI in a given country at 38.6%.

**Conclusions:**

Political polarization can contribute to shaping negative attitudes towards vaccination through policy distortion, undermining the effectiveness of compliance against regulation, undermining trust in science and increasing the public's susceptibility to conspiracy theories.

## Introduction

Political polarization has been increasing in many countries around the world in recent years, largely owing to the rise of populist and right-wing governments.^1^ Political polarization may result in increased confirmation bias and, consequently, the strengthening of disinformation mechanisms^2^ or policy distortion,^3^ thus, undermining the effectiveness of regulations, including those related to public health. This raises the question of whether such specific contexts influenced how the governments responded to the coronavirus disease 2019 (COVID-19) pandemic and determined whether the vaccination programmes in these countries were effective or not.

This study investigates the relationship between the level of political polarization and vaccine behaviour and vaccine acceptance. Based on data from the latest round of the European Social Service (ESS) survey, this study involved a comparison among 23 countries with different levels of political polarization and different COVID-19 vaccination declarations and declared vaccination intent.

Over the years, some studies have highlighted the relationship between vaccine attitudes and political identities, revealing the higher propensity for vaccine scepticism among far-right supporters,^4^ voters favouring populist parties^5^ and supporters of conservative parties.^6^ However, using the latent class analysis method, Hornsey et al.^7^ showed that vaccine scepticism is associated not only with the far-right but also the far-left and liberal views. Analysing the case of France, Ward et al.^8^ indicated that the number of vaccination sceptics was the highest among those unaffiliated with any political party.

The present study explores the relationship between political factors and vaccine attitudes, using the perspective of political polarization rather than that of support for one party or the other. The COVID-19 pandemic-related research has provided evidence that polarization may affect health crisis management and the effectiveness of regulations.^9–12^ However, there are few studies that investigate the impact of political polarization on the effectiveness of COVID-19 vaccination programmes. Some available studies point to a relationship between the level of polarization and COVID-19 vaccine attitudes. For example, vaccine attitudes in Brazil, a country with strong internal conflicts, were associated with political polarization.^13^ A link between political polarization and attitudes towards COVID-19 vaccination was found in the USA too.^14^

## Methods

In this study, political polarization is defined as a division within society based on political party preferences and the degree of identification with specific political forces, rather than on ideological differences. This definition largely overlaps with affective polarization,^15^ which is characterized by (i) a link between partisan identity and social identity,^16^ (ii) a negative bias towards the out-party and a positive attitude towards the in-party^17^ and (iii) a strong bimodality.^18^ In this approach, political polarization is shaped primarily by the gap between government supporters and opponents rather than the intensity of political conflicts. In our study, we use Veronika Patkós’^18^ Partisan Polarization Index (PPI) to estimate the level of political polarization:

$$\mathrm{PPI}=\;\frac{\mathrm{Sgov}}{\mathrm{Sopp}},$$

where PPI: Partisan Polarization Index; Sgov: average satisfaction with the government (government supporters); Sopp: average satisfaction with the government (opposition supporters).

The PPI aims to measure the gap between satisfaction with the government expressed by supporters of the ruling parties and that expressed by supporters of the opposition parties. The underlying idea is that satisfaction with the government is identity-specific in polarized societies, while in less polarized societies, it is, to an extent, based on other more content-oriented factors. In the latter case, some ruling party supporters can express lower satisfaction with the government, while part of the opposition may express higher satisfaction, consequently reducing the gap between the aggregate satisfaction levels and lowering the PPI.

We decided to use the PPI for two reasons. First, the PPI, based mainly on attitudes towards the government and sympathies towards the ruling and opposition parties, works better as an index indicating the level of political polarization in the European multiparty political system. Secondly, as pointed out above, research on the relationship between political ideologies and attitudes towards vaccination is sometimes non-conclusive, and the relationship is often linked to a specific political context. As indicated above, the phenomenon of polarization is different from supporting one ideological option or another because it is based on the difference between the supporters and opponents of the government.

The analysis carried out for this study was based on data from the ESS. Attitudes towards vaccination were analysed using the *getavc19* variable (‘Whether the respondent get vaccinated against COVID-19 with the vaccine approved by the national regulatory authority in the country?’). The respondents who answered this question with ‘Yes, I will’ (vaccine intention) and ‘Yes, I already have’ (vaccine behaviour) were included in the analysis. The variable determining the ratio of people declaring vaccination and vaccination intent to the general population is hereinafter referred to as ‘vaccination’.

The *getavc19* variable has already been used in other vaccine attitude analyses.^19^ When our study was being conducted, no *getavc19* variable data were available for France. This gap was supplemented by the estimated vaccination coverage in the adult population at the time the ESS was conducted. The estimation was based on the European Centre for Disease Prevention and Control (ECDC) with adjustments for differences between the declarations in the ESS and the ECDC. In the analysis, we assumed that the gap between ECDC and ESS data for France is the EU average (see table 1). The ECDC result for France multiplied by the average ESS/ECDC factor for all countries translates into a 96% vaccination rate.

**Table 1:** Country-wise analysis parameters

ID	Country	Vaccination (ESS: behaviour and intentions)	Vaccination level (ECDC)	ESS/ECDC	PPI	No party identification
1	Austria	0.89	0.81	1.1	1.64	0.32
2	Croatia	0.7	0.64	1.09	2.15	0.67
3	Czechia	0.72	0.73	0.99	1.7	0.72
4	Estonia	0.8	0.73	1.1	1.34	0.65
5	Finland	0.96	0.86	1.12	1.41	0.42
6	France	0.96^a^	0.87	–	1.54	0.61
7	Germany	0.92	0.81	1.14	1.25	0.54
8	Greece	0.88	0.77	1.14	2.6	0.72
9	Hungary	0.77	0.7	1.1	2.62	0.64
10	Iceland	0.97	0.91	1.07	1.58	0.53
11	Italy	0.96	0.84	1.14	1.4	0.77
12	Lithuania	0.79	0.77	1.03	2.06	0.71
13	Montenegro	0.83	0.83	1	1.32	0.73
14	Netherlands	0.92	0.78	1.18	1.35	0.71
15	North Macedonia	0.76	0.4	1.9	2.31	0.48
16	Norway	0.96	0.89	1.08	1.13	0.37
17	Portugal	0.96	0.93	1.03	1.34	0.53
18	Serbia	0.71	0.62	1.15	3.59	0.59
19	Slovakia	0.7	0.49	1.43	2.5	0.76
20	Slovenia	0.64	0.65	0.98	3.18	0.72
21	Spain	0.97	0.85	1.14	2.4	0.51
22	Sweden	0.96	0.84	1.14	1.61	0.32
23	Switzerland	0.82	0.63	1.3	0.99	0.55
	Mean	0.83	0.73	1.11	2.08	0.59

a No data on ESS, estimation based on ECDC.

Respondents were divided into two groups to calculate the PPI: the government supporters and the opposition supporters. The former were people who declared feeling close to a party (the *clsprty* variable) and simultaneously confirmed feeling closer to the government parties (the *prtcl* variable). The latter—opposition supporters—were people who declared being closer to a party (the *clsprty* variable) and simultaneously indicated feeling closer to the opposition parties. The level of satisfaction with the government was calculated based on the *stfgov* variable: ‘Now thinking about the [country] government, how satisfied are you with the way it is functioning?’. The ESS study coincided with a government change in Germany, which was considered in the development of the PPI.

Patkos uses two PPI in her analysis—one based on satisfaction with the government (*stfgovI* variable) and the other, PPI (voters), on declarations about voting in the last election (*prtvt* variable). In our analysis, we use the former index as better suited to assessing the level of polarization as a phenomenon related to strong identification with a particular political party (see table 1).

Further analyses included respondents who answered in the negative to the question on feeling closer to a party (the *clsprty* variable) and provided the basis to produce another variable related to less politically engaged persons. The factor of persons declaring not feeling close to a specific party for individual countries is shown in table 1 (no party identification).

The effect of the PPI on the variability of vaccination declarations and intent across countries was verified based on a linear regression model. Based on Tukey’s rule that considers outliers to be those whose value exceeds 1.5 times the interquartile range,^20^ excluded Bulgaria and Poland from the analysis as an outlier for the PPI variable and Poland as one for the vaccination rate. The variable ‘non-party identification’ (based on the *clsprty* variable) does not have outliers. In this study’s analysis, Poland’s PPI proved to be extremely high, much higher than for other countries; a possible reason is the overrepresentation of the supporters of the main opposition party in Poland’s sample. Another possible explanation is the absence of a link between political polarization and vaccine attitudes in Poland. At the time of the conduct of the survey in Bulgaria, several technical governments were formed consisting of non-political experts, making it more difficult to estimate the level of political polarization. Then data containing 23 countries (excluding Bulgaria and Poland) were admitted to the final sample and revealed a linear relationship between vaccination and PPI.

## Results

Table 1 reveals that the vaccination variable for the entire group of countries ranged from 0.64 (Slovenia) to 0.97 (Iceland and Spain), and the PPI ranged from 1.32 (Montenegro) to 3.59 (Serbia). Considering this range, countries with vaccination between 0.6 and 0.8 could be described as those whose respondents were less likely to declare vaccination or vaccination intent, while those with a score above 0.8 comprised countries where respondents had a more positive attitude towards the COVID-19 vaccines.

Patkós,^18^ whose methodology for calculating the level of political (partisan) polarization was applied in this study based on several ESS rounds, identified Spain, Croatia, Greece and Hungary to be among the most polarized countries. The analysis carried out for our study found these countries to be more polarized than others as well. Additionally, in our study high political polarization was observed in North Macedonia, Slovenia and Serbia.

The first step of the analyses evidenced PPI’s significant influence on vaccination, expressed as a linear relationship stating that 38.6% (*R*^2^) of the variability of vaccination in European countries resulted from the polarization level measured with the PPI based on declared closeness to the ruling or opposition party. The linear regression is statistically significant (*P* = 0.002). The Kolmogorov–Smirnov test for the distribution of residuals yielded a value of *P* = 0.2 and the Shapiro–Wilk *P* = 0.4, which allows us to positively validate the model.

The above result suffers a major limitation due to the percentage of people not declaring closeness to a party (the *clsprty* variable) being significant and varying from country to country (see table 1). These people can be considered less politically engaged and thus not building polarization. Therefore, we decided to consider the share of these individuals in the population of each country as another variable that can model the level of vaccination. The lack of correlation between the PPI and the level of people not declaring closeness to a party (Pearson Correlation *P*  = 0.732) enables us to employ both variables in multiple regression.

Including the additional factor increased the predictability of the variability of vaccination in a country to 44.8% (Adjusted *R*^2^). The result of this regression (*y* = 1.167 − 0.084 × X1 −0.272 × X2) indicates that as the proportion of people not declaring closeness to a party increases, the level of vaccination decreases. The second factor, the proportion of people not expressing closeness to any party, affects the dependent variable such that with each percentage of people not engaged, the level of vaccination decreases by 0.27%. In the same regression, as the PPI increases by 1 point, the probability of vaccination decreases by 8%. Beta standardized indices show that the PPI affects vaccination more strongly (Beta standardized = −0.5, significance = 0.004) than the lack of closeness to a party (Beta standardized = −0.3, significance = 0.047). The regression is statistically significant (*P* = 0.001) and the distribution of residuals from the multiple regression also demonstrates a normal distribution: the Kolmogorov–Smirnov test for the distribution of residuals yielded a *P* value of 0.2, and Shapiro–Wilk yielded a *P* value of 0.4, allowing us to positively validate the model.

## Discussion

The present study shows that political (partisan) polarization can emerge as an important factor affecting vaccine behaviour and attitudes. As mentioned, political polarization can lead to policy distortion,^2^ which occurs when political decisions reflect the views of the electorate and are oriented towards securing a quick political gain. This problem intensifies in crises when policymakers must take more radical actions, including ones not necessarily approved by the public.^11^

Concerning the COVID-19 vaccination programmes, an example of a public policy that raised particular concern was the introduction of COVID-19 passes. Italy was among the first EU member states to implement restrictions for the unvaccinated. In our study, Italy is a highly polarized country with many people declaring that they were either vaccinated or willing for the same. Although protests were raised against the COVID-19 passes, their introduction was met with a political consensus and approval from the majority of society, neither of which would be likely in a more polarized country.^21^ Another example is Slovenia, classified as a country of high polarization. COVID-19 passes were introduced there in the autumn of 2021; however, this decision inspired mass protests (referred to as ‘the largest ever protest in Slovenia’^22^). Eventually, the Slovenian Constitutional Court overturned these measures in April 2022. It is worth adding that the COVID-19 passes were introduced in Slovenia by a decree rather than a law passed by the parliament, which was criticized by the opposition and non-governmental organizations.^23^ According to both the ESS results and ECDC data, Slovenia is among the EU member states with the lowest COVID-19 vaccination coverage.

Political polarization may also negatively impact the effectiveness of regulations. This is related to what is known as polarized political trust,^24^ a situation when trust in certain institutions is essentially dictated by one’s attitude towards the government. In highly polarized societies, people may be unwilling to trust various social institutions that they perceive as an extension of the state and the government. The implementation of certain policies may face significant obstacles, given that non-compliance with government policies will, for many, be tantamount to contesting those currently in power. Concerning COVID-19 vaccines, it was in Serbia that vaccine scepticism was associated with the lack of trust in the government and health institutions.^25^ The analysis carried out for this study showed Serbia as an example of a highly polarized country, marked by vaccine aversion and low vaccination coverage.

Societies in which trust in science and scientists is high are more vaccine-positive than sceptical ones.^26^ Political polarization can contribute to increased levels of scepticism towards science for two reasons.^27^ First, in polarized societies, decisions can be made based on statements from political leaders rather than expert recommendations. This top-down mechanism relies on the power of identification by the proponents of a particular political camp with its leaders. As Ward et al.^28^ argued, ‘When political leaders take a public stance on vaccines, they signal to the public that the political dimensions of their identity can (and should) affect their judgment on this issue’. Given that this relationship is stronger in highly polarized societies, the authority of politicians can mean more to the public than that of scientific experts. Secondly, high polarization is associated with political populism.^1^ In populist narratives, science can be shown as an expression of the elite’s worldviews, while its denial can be perceived and presented as opposition to the establishment.^27^ In a situation of high polarization, narratives opposing science are more likely to emerge and be accepted by the public. In the context of vaccination, high polarization can significantly weaken the position of scientists and experts in society.

There is also a link between conspiracy thinking and political polarization, attributable to three reasons. First, populist narratives, based on anti-establishment resentments that can contribute to the popularity of conspiracy theories, are much more effective in highly polarized societies.^27^ Secondly, conspiracy theories can be popular in a situation of low trust in science, which, as mentioned, can also be due to political polarization. Thirdly, high polarization influences debates on social media that have emerged as a key source of conspiracy theories. In other words, the higher the polarization, the greater the tendency of online communities to close themselves in filter bubbles, which increases both the pace of the spread of conspiracy theories and their reach.^29^ Conspiracy thinking negatively affected preventive behaviour during the COVID-19 pandemic, including behaviour related to vaccination.^30^

A correlation between conspiracy thinking and vaccine attitudes was identified in another analysis, too, with the same ESS database as the one used for the present study.^19^ Its authors also included Slovakia, North Macedonia, Croatia and Slovenia, all recognized as highly polarized by our study, among countries with a higher index of belief in conspiracy theories. Another study,^31^ also based on ESS data, described a link between conspiracy thinking and vaccine uptake, satisfaction with how health services handled the pandemic and adherence to restrictions.

Our analysis is limited by the aforementioned significant percentage of people who do not declare closeness to a party. It may indicate the role of disenchantment with politics in shaping attitudes toward vaccination. Our results are in line with a study by Ward et al.,^8^ concerning France. In this study, non-partisans were the largest vaccine-hesitant group. As the authors argue, explaining vaccine choices by the influence of political identity formed as a result of high polarization may be limited, and issues such as the level of institutional trust may play a larger role in the process. Indeed, a decline in institutional trust can lead to disenchantment with politics and, as many studies indicate, can increase vaccine scepticism. Spält et al.^32^ on the other hand, based on research conducted in Spain, argue that anti-elite worldviews (anti-expert and conspirational worldviews) play a greater role than partisanship (understood as feeling closest to a particular party) in shaping misperceptions about vaccination.

It is worth noting here that the multiple regression indicated that polarization is a stronger factor affecting vaccination coverage than the lack of political commitment. From our perspective, however, the fact that lack of political commitment is strongly correlated with vaccine hesitancy is an important finding indicating the existence of different trajectories of the phenomena we studied. Indeed, the impact of political engagement can affect attitudes towards vaccination in different countries in different ways. In one context, political polarization may be a stronger influencing factor, while in another disenchantment with politics may be a factor. To delve deeper into this thread, however, would require additional analyses beyond the thematic scope of our analysis.

## Conclusions and limitations

The analysis carried out for this study revealed that political context was an important factor that shaped attitudes towards COVID-19 vaccination. Our approach, based on the impact analysis of political polarization, shows that differences between the supporters and opponents of the government may be an important factor shaping vaccine attitudes. The greater the discrepancy in the evaluation of the government’s actions, the less positive the vaccine attitudes expressed as a vaccination or vaccination intent declaration. Political polarization may give rise to or strengthen several phenomena affecting vaccine attitudes, such as policy distortion, the effectiveness of regulations, polarized political trust, erosion of expert authority and an increased tendency towards conspiracy thinking.

**Figure 1:**
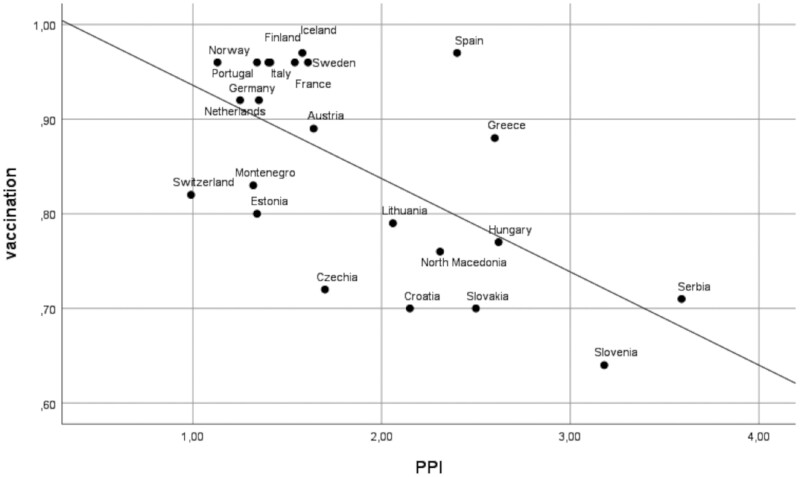
Visualization of linear regression

The analysis carried out for this study has certain limitations. First, the use of the ESS database meant that there was reliance only on one variable related to vaccine attitudes, which imparted to our study the character of exploratory research. More in-depth research on the relationship between vaccine attitudes and political polarization is required, using more sensitive measurement techniques. Secondly, our study does not go into the differences between countries regarding the extent to which political polarization affects vaccine attitudes. Figure 1 indicates potential similarities between countries and differences between groups of countries. Conducting additional analyses and describing them adequately is unfortunately beyond the limits of the article. More case studies are needed to illustrate how local factors contribute to the dynamics of the relationship highlighted in our study. Noting the differences in the respective contexts is also essential because, as we have indicated several times, in the database we analysed, a relatively large proportion of respondents do not feel affiliated with any party. Although such attempts have already been made,^28^ disenchantment with politics should be further researched in terms of vaccine attitudes, as well as in the context of the relationship of this phenomenon to political polarization. Finally, it should be noted that the ESS survey was conducted at a very specific time in which the operation of certain attitudes, due to the health crisis, may have been different from the non-crisis context.

## Funding

Funding support for this article was provided by the Research University Excellence Initiative.

*Conflicts of interest*: None declared.

## Data Availability

The data that support the findings of this study are available from European Social Survey: https://doi.org/10.21338/ess10e03_1.
